# The Calorimetric Detection of Excited States*

**DOI:** 10.6028/jres.080A.042

**Published:** 1976-06-01

**Authors:** James B. Callis

**Affiliations:** Department of Pathology, University of Washington Medical School, Seattle, Washington 98195

**Keywords:** Absolute quantum efficiency, absolute quantum yield, calorimetry, luminescence, photoacoustic spectrometer, piezocalorimeter, transducers, triplet formation

## Abstract

Calorimetric techniques offer the photophysicist and photochemist the opportunity to measure a number of parameters of excited states which may be difficult to obtain by other techniques. The calorimetric strategy seeks to measure the heating of a sample resulting from radiationless decays or chemical reactions of excited states. Heating is best measured through volume and pressure transducers, and four calorimeters based on these are described. With calorimetric instrumentation one can perform measurements on samples in the gas, liquid and solid phases over a wide temperature range. Moreover time dependent processes with time constants ranging from microseconds to seconds are amenable to study. Examples of the application of calorimetric techniques to the determination of quantum yields of fluorescence, triplet formation and photochemistry are given.

## 1. Introduction

In understanding the nature of excited electronic states it is well appreciated that a wide variety of parameters are accessible to photometric measurement. Among them are the absorption spectrum of the excited state, the excitation spectrum, and the lifetime. If the excited state decays by emission of photons, one can also measure the spectral distribution of emission, the polarization, and the quantum yield of emitted photons. Unfortunately, it is not so generally appreciated that a number of parameters of an excited state are also accessible to calorimetric measurement. The calorimetric strategy seeks to measure how much of the light absorbed by a sample is converted to heat energy by radiationless processes and/or photochemistry. Parameters which can be measured include: the lifetime of an excited state, the excitation spectrum for a particular process, the energy yield of a radiationless process, and the enthalpy of a photochemical process.

A simple example illustrates how calorimetric measurements can complement photometric measurements. Most air saturated liquid solutions of luminescent organic molecules exhibit only fluorescence at room temperature. By measuring the ratio of the heating of the fluorescent substance in response to photoexcitation to that of a non-fluorescent but equally absorbing substance, one obtains the energy yield of radiation-less processes *Y_h_* [1, 2].^1^ In the absence of photochemistry, the fluorescence energy yield is the complement of *Y_h_*, and the quantum yield of fluorescence Φ*_f_* is related to it by the formula

$$\Phi_{f}=\left({\bar{v}}_{a}/{\bar{v}}_{f}\right)\left(1-Y_{h}\right)$$ (1)

where ${\bar{v}}_{a}$ and ${\bar{v}}_{f}$ are the average frequencies of absorbed and emitted photons respectively. Calorimetric quantum yields determined in this manner [1, 2, 3] are among the most precise and accurate reported in the literature, and in addition provide a valuable independent technique for verifying the many assumptions that go into the derivation of quantum yields from photometric measurements [4, 5]. Despite these advantages, very few quantum yields have been measured calorimetrically. This unfortunate situation exists because most workers in the field are unfamiliar with calorimetric techniques, and moreover, the measurements are time consuming and tedious to perform. It is the purpose of this paper to point out some recent work which shows that using capacitor microphones and piezoelectric crystals as heat flow transducers, calorimetry can be a rapid, simple and sensitive technique for use in measuring a wide variety of parameters of excited states. Also, samples in the gas, liquid and solid phases, including thin films and monolayers are amenable to study.

## 2. Types of Calorimeters Suitable for Measuring Photochemical and Photophysical Processes

The most obvious approach for detection of heat production is to use a conventional calorimeter based upon the usual temperature sensors: thermistors and thermopiles. We will not discuss these instruments here because they have already been reviewed [3, 4, 5] and because they have inherent disadvantages in sensitivity, risetime and speed at which measurements can be made. Instead, we will concentrate on alternative strategies for measuring heat flow based on volume and pressure changes produced in the sample.

In general, addition of an amount of heat *dQ* to the sample gives rise to both a pressure change *dP* and a volume change *dV* according to the thermodynamic relationship [6]

$$dQ=\frac{C_{P}dV}{\alpha V}+C_{v}\frac{\beta }{\alpha }dP$$ (2)

where *C_P_* and *C_v_* are the heat capacities at constant pressure and constant volume, *V* the volume of the solution, *α = V*^−1^(∂*V*/∂*T*)*_P_* is the coefficient of thermal expansion, and *β* = *−V*^−1^(∂*V*/∂*P*)*_T_* is the compressibility of the sample. Three of the calorimeters we will describe use the capacitor microphone to detect volume changes in the sample. In all of these devices the sample is enclosed in a cell with rigid walls. One of these walls, generally normal to the direction of propagation of exiting light is replaced by the compliant diaphragm of a capacitor microphone. Because of the diaphragm, a second relationship between *dP* and *dV* must be simultaneously obeyed:

dP=κdV/V. (3)

We have assumed that the changes are small so that a linear relationship exists, and *κ*, the linear constant, is approximately given for a cubic cell by *k*/*ℓ* where *k* is the force constant for displacing the diaphragm, and *ℓ* is the linear dimension of the cell. Combining eq (2) and (3) we have

$$dV=\alpha VdQ/\left(C_{P}+C_{V}\kappa \beta \right).$$ (4)

Equation (4) shows that: (a) the volume change is independent of any gradients in the distribution of energy in the cells; (b) the largest volume changes are obtained for samples which have the highest values of *α* and the lowest heat capacities (gases give the largest signals; water gives low signals); (c) the compliance of the diaphragm must be kept low enough so that the product *κβ* is as small as possible.

The capacitor microphone for measuring the volume changes consists of the diaphragm and a stationary electrode spaced a small distance apart; together they form a capacitor whose capacitance *C* is given by

C=ϵA/x (5)

where *ϵ* is the dielectric constant, *A* is the area of the electrodes, and *x* is the spacing between them, and edge effects were neglected. If the change in spacing of the electrodes is small enough, the capacitance will be related to it linearly. Several methods have been used successfully for the detection of the capacitance change. In the most common method [7], the microphone is polarized either with an externally supplied dc voltage or by placing an electret [8] in the gap between the electrodes; when the capacitance changes, charge flows to maintain a constant voltage. The charge flow is then converted to a voltage change by a charge sensitive pre-amp [9]. Capacitance changes can also be detected with an ac bridge circuit [10]. Even with simple detection systems, a displacement of the diaphragm of the order of one angstrom is easily measured with a bandwidth of 10 kHz [6]. The frequency response of the microphone is linear over a very wide range; the upper limit is set by the lowest mechanical resonance of the diaphragm (10–20kHz), and the lower limit is determined by the low frequency cut-off of the electronics or the thermal instabilities of the system.

With these fundamental considerations in mind, we may now discuss three types of calorimeters based upon the use of capacitor microphone pressure transducers: the “acoustic spectrophone” for study of gas phase systems, the “optoacoustic cell” for study of thin films and thin sections of solid materials, and the “flash calorimeter” for studies of liquid samples.

The acoustic spectrophone has been widely used as a technique for infrared gas analysis [12] and for measuring vibrational relaxation rates [13]. A typical acoustic spectrophone [11] consists of a cylindrical cell with transparent windows at each end to allow radiation to enter and leave; the microphone is generally mounted on the cylindrical wall with the diaphragm normal to the direction of propagation of the excitation beam. Often, a regulated leak is provided so that long term pressure drifts can be relieved.

The design of acoustic spectrophones is discussed in detail by Rosengren [14], Parker and Ritke [11] and Kerr and Atwood [15]. The ultimate limit of signal to noise arises from the Brownian motion of the gas molecules. Present designs can come within two orders of magnitude of this limit at reasonable gas pressures. The present limitations arise from noise in the detection electronics, temperature instabilities (especially at low frequencies) and spurious contributions to the signal arising from gas adsorbed on the windows [14]. Even with these limitations, energy inputs as small as one microwatt may be easily detected. Parker has shown [11] that the rise time for the volume change is set by the lowest acoustical resonance frequency in the cavity whose frequency is given by *c*/2*l*, where *c* is the speed of sound in the gas, and *l* is the length of the cavity.

A device similar to the acoustic spectrophone has been developed by Rosencwaig [17] for the study of radiationless processes and photochemistry in thin films of solids, and semisolid materials such as crystals, evaporated films, powders, gels, thin layer chromatography plates, and even thin layers of tissue. The photoacoustic cell is quite similar to the acoustic spectrophone of Parker, except it is much shorter in cavity length. The thin sample is simply mounted flush with the exit window, and the cell is filled with a suitable gas which is transparent to the exciting radiation.

The origin of the photoacoustic effect is believed to be as follows [11, 18]: irradiation of the sample with pulsed or chopped steady state light results in a localized heating due to non-radiative decay processes by the excited states. Some of the heating is transferred via diffusion from the solid to a thin boundary layer of gas adjacent to the solid. Adiabatic expansion of the gas then gives rise to a pressure wave in the gas which is detected by a capacitor microphone. A quantitative version of this process has been proposed [11, 18]. Like the acoustic spectrophone this device can easily detect microwatts of absorbed radiation, and the risetime is set by the transit time for sound in the cavity.

**Figure 1:**
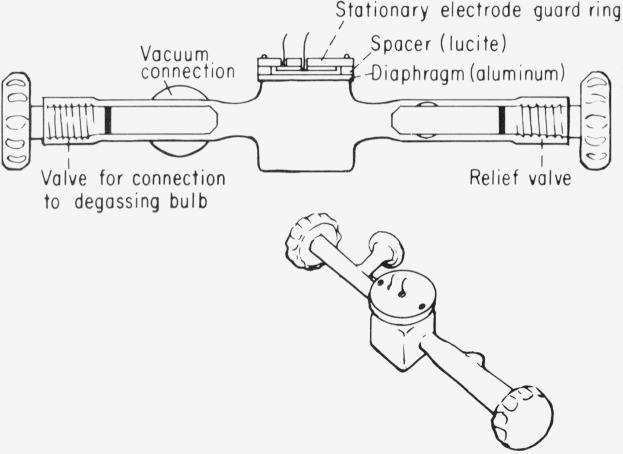
Flash calorimeter schematic.

The third device based on the use of the capacitor microphone is the flash calorimeter of Callis, Gouterman and Danielson [6]. A schematic of the device is given in Figure 1. The sample cell is constructed from 25×25 mm square Pyrex^2^ tubing. Joined to the sides are two 4-mm Pyrex-Teflon vacuum stopcocks. One of these provides connection to a vacuum line, allowing for introduction of degassed samples. The other stopcock opens into a small relief reservoir. It provides a controlled leak so that liquid can slowly enter or leave the cell, thus preventing slow pressure changes that would occur in a completely closed cell subject to temperature drifts. To allow use of a circular diaphragm a short piece of 25-mm Pyrex tubing is fused to the top of the cell. The diaphragm is made from 1 mm aluminum, which is sufficiently thick so that it does not deform permanently under vacuum, yet not so thick that the product *κβ* exceeds unity. The microphone consists of the diaphragm, an epoxy glass circuit board spacer, and a stationary electrode-guard ring assembly fabricated from brass and epoxy glass circuit board material. The spacer ring is ground down until the gap between diaphragm and stationary electrode is approximately 0.25 mm and the entire assembly is potted with epoxy cement. The cell is mounted in a brass block which is temperature stabilized by means of a thermoelectric heat pump. The entire assembly is mounted in an acoustically shielded aluminum box which rests on a vibration isolation table.

As with other devices based on the use of microphones, the rise time of the flash calorimeter is determined by the transit time for sound in the cavity. This limits the use of the instrument in kinetic studies to phenomena with time constants longer than 100*μ*s. The sensitivity of the flash calorimeter is quite remarkable; a displacement of the diaphragm of 10^−8^ cm is easily detected with a bandwidth of 10 kHz. For ethanol this corresponds to a temperature rise of ~ 10^−6^ °C or an energy input of ~ 64*μ*J. The limits of detection at high frequencies are largely due to the noise in the preamplifier stage; we are presently many orders of magnitude above the limit imposed by the thermal noise of the sample.

We now describe an instrument which is particularly well suited for measurement of radiationless processes in bulk solids. This device, the piezoelectric calorimeter, uses a piezoelectric crystal to measure pressure changes in the sample. In certain crystals and polycrystalline materials with assymetric charge distribution, an applied pressure results in displacement of the positive and negative charges relative to each other. The displacement of the charges can be measured by applying electrodes to the surfaces and measuring the potential difference between them caused by charge migration. Piezoelectric crystals are available which are rugged, inexpensive, and linear and which will perform at temperatures from 4 K to 700 K.

**Figure 2:**
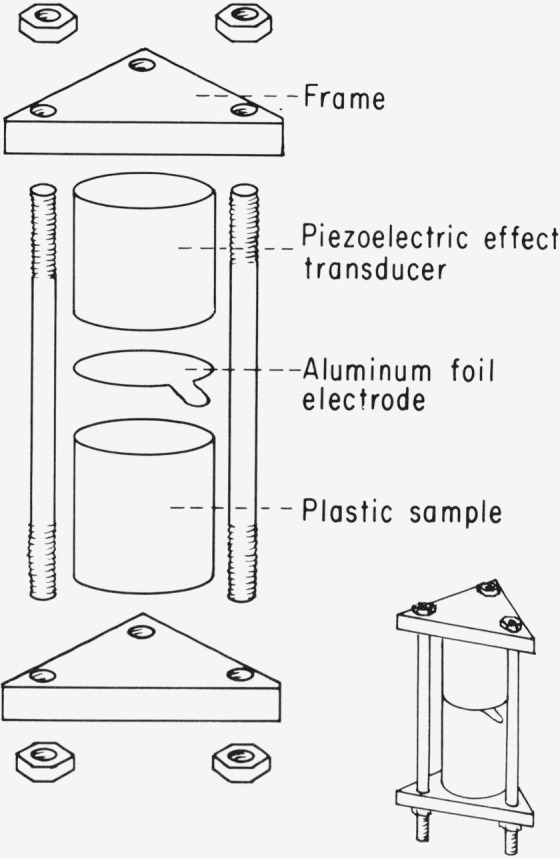
Piezocalorimeter schematic.

A simple piezoelectric calorimeter is shown in figure 2. It consists of a cylindrical sample (we have used polymethyl methacrylate samples in which the organic compound of interest is dissolved), and a cylindrical piezoelectric transducer of the same radius as the sample. The transducer and sample are clamped rigidly together by an aluminum frame.

The piezoelectric crystal may be conveniently thought of as measuring the pressure generated by heating at constant volume. Equation (4) then becomes

$$dP=dQ\alpha /\beta C_{V}.$$ (6)

The open circuit voltage response to a pressure change is given by:

E=gtdP (7)

where *g* is the open circuit voltage constant in units of m^2^/C, *t* is the thickness of the crystal in m, and *E* is the voltage produced [7]. The simplest method for operating these devices is to use a charge sensitive preamplifier, as with the capacitor microphone. The upper end of the frequency response will then be limited by the lowest acoustic resonance, while the lower end will be limited by the low frequency response of the preamplifier.

The response of the piezoelectric calorimeter to an infrared heating flash shows characteristics similar to that of the flash calorimeter. The ring period is shorter due to the higher speed of sound in the cavity, but is not damped as effectively. With the present apparatus we can easily detect a step rise of *E* ⩽ 10^−3^V which corresponds to a pressure change of 0.073 dyn/cm^2^ for a lucite rod of 1 cm thickness, which in turn corresponds to a temperature change of 6.7 × 10^−7^ °C. For our samples we find that *dQ* ~ 4.7 *μ*cal is easily detected.

In addition to the calorimeter, the measurement of calorimetric parameters also requires a light source, a means for selecting a bandwidth of the radiation, and suitable signal processing electronics. The ideal light source for calorimetry must be of high intensity, stable with time, and of high spectral purity. Clearly the laser approaches most closely this ideal, and a number of authors have employed both cw and pulsed lasers. For excitation spectrum studies a Xenon arc lamp-monochromator combination possesses the one advantage of infinite wavelength tunability and the capability of ultraviolet irradiation. Since the calorimeters are ac coupled, the light source must be intensity modulated. For a cw light source, a simple and common method to achieve intensity modulation is with a mechanical chopper. In this case, the most attractive form of signal processing is to use a phase sensitive amplifier, operated in synchrony with the chopper. If all of the heating from the sample appears with a rate constant greater than the chopping rate, then all of the signal will be in phase with the chopper. However, if some of the heating arises from processes which have rate constants comparable to or less than the chopping rate, then some of the heating will be out of phase. Quantitation of these effects leads to methods for measuring rates of relaxation of excited states and the yield of heating of a particular process. The disadvantages of modulation of a cw source and phase sensitive detection is that the signal decreases linearly with the frequency, and that a series of measurements must be made at a number of different frequencies if the order of the kinetics is to be specified.

For the measurement of the time dependence of the heating, the use of a pulsed light source is advantageous. Large amounts of energy can be output in very short times, thus providing a good signal to noise ratio for fast time dependent processes, as well as giving the heating curve directly. This technique requires some method for transient recording and signal averaging is often desirable. A potential problem with pulsed excitation is that it strongly excites the lowest cavity resonance of the chamber. The resultant oscillation is superimposed on the signal and often obscures the heating curve at short times.

## 3. Applications of Calorimetric Techniques

### 3.1. Fluorescence Quantum Yields

In the introduction, we noted the advantages of the calorimetric strategy for determination of quantum yields. Thus far, all of these measurements have been done on dilute liquid solutions which are easily done photometrically. In contrast determination of quantum yields from thin layers of powdered samples is a very difficult procedure to perform photometrically [19, 20]. Some pioneering work by Rosencwaig [17, 21] illustrates how the optoacoustic spectrometer can be used to advantage on these systems. A number of trivalent rare earth ions show narrow absorption bands in the visible and ultraviolet which arise from inner shell f–f* transitions. Some of these levels exhibit fluorescence, and the fluorescence quantum yield of a particular level exhibits a marked dependence upon which level is excited [22]. Rosencwaig studied the photoacoustic excitation spectrum of samples of powdered holmium oxide. The first sample contained cobalt and fluorine impurities in sufficient quantity to quench all of the luminescence. He found that the photoacoustic spectrum, when corrected for the wavelength dependence of the relative output of the excitation beam, corresponded well with the observed absorption spectrum. In contrast, the photoacoustic spectrum of pure Ho_2_O_3_ exhibited a spectrum in which some of the lines were greatly diminished in intensity; the latter of course involve excited states which decay by radiative processes. The information contained in these two scans, together with lifetimes and luminescence spectra taken by exciting at each major absorbance would provide, for the first time, a complete picture of the dynamic interrelationships of these excited states.

### 3.2. Quantum Yields of Triplet Formation

A knowledge of quantum yields of triplet formation is important in understanding the intersystem crossing process, and also in understanding the many photochemical reactions and energy transfer process which take place through the intermediacy of a triplet state. A number of ingenious methods exist for determination of triplet yields [23] but all are based on the use of various assumptions which are difficult to verify experimentally. The calorimetric strategy offers an attractive alternative to the other techniques, and also provides an independent check on the assumptions used in the other methods.

The calorimetric method is based on the fact that the lifetime of the lowest triplet state of an organic molecule is long compared to that of its excited singlet states. Thus, we expect that a delta function excitation pulse will produce fast heating due to relaxation of the singlet states, and slow heating due to relaxation of the triplet states. The heating may then be partitioned into

$$Q_{\text{tot}}=Q_{\text{fast}}+Q_{\text{slow}}.$$ (8)

It has been shown (6) that Φ*_t_* can be obtained from the relation:

$$\Phi_{t}=Q_{\text{slow}}\left[h{\bar{v}}_{in}-\Phi_{f}h{\bar{v}}_{f}\right]/Q_{\text{tot}}E_{t}$$ (9)

where Φ*_f_* and Φ*_t_* are the quantum yields for fluorescence and triplet yields, ${\bar{v}}_{f}$ and ${\bar{v}}_{in}$ are the average fluorescence and absorbed frequencies, and *E_t_* is the energy of the triplet state.

**Figure 3:**
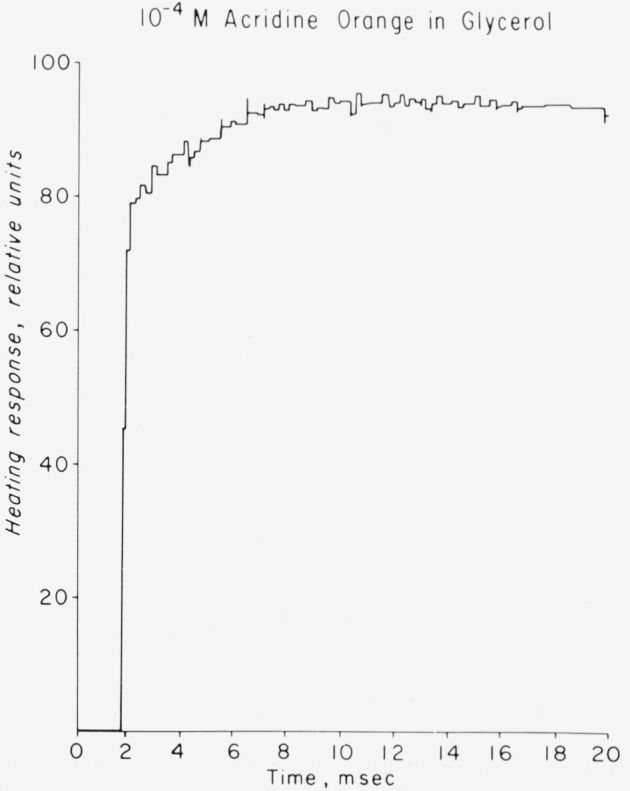
Calorimetric response of degassed acridine orange in glycerol at room temperature.

**Figure 4:**
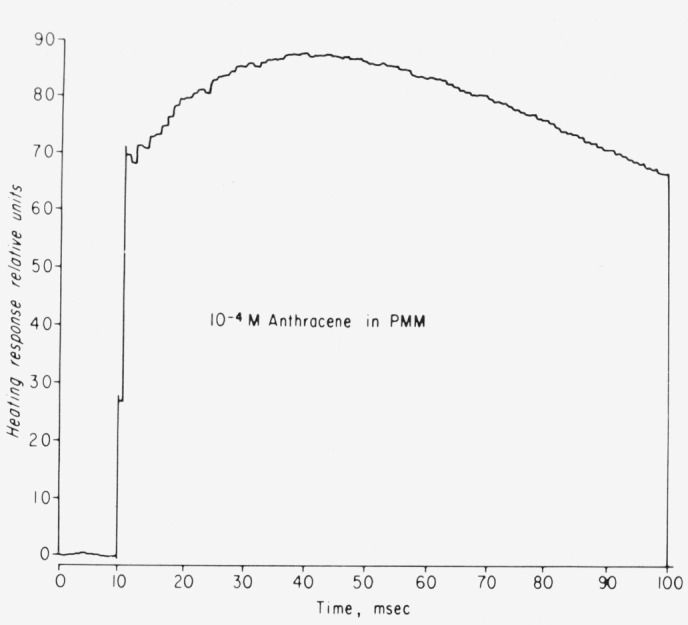
Calorimetric response of anthracene in polymethyl methacrylate at room temperature.

As an example of the use of the flash calorimeter we show the heating response of 10^−4^ *M* acridine orange to flash excitation in figure 3. The results are the average of 100 flashes. Clearly, there are two kinds of heating, as expected. Furthermore, the observed heating obeys the expected [6] time dependent form,

$$Q\left(t\right)=\left[Q_{\text{fast}}+Q_{\text{slow}}\left(1-e^{k_{p}^{*}t}\right)\right]e^{-t/RC}$$ (10)

where $k_{P}^{*}$ is the triplet decay time, and *RC* is the decay time of the ac coupled electronics. For comparison, in figure 4 we show the heating response of 10^−4^*M* anthracene in polymethyl methacrylate as studied with the piezoelectric calorimeter. It also shows the same type of heating response as predicted by eq (10). The value Φ*_t_* = 0.72 for anthracene in plastic compares well with that determined in mineral oil by other methods [23].

### 3.3. Applications to Photochemistry and Photobiology

In addition to heating from radiationless transitions, one may also detect heating from photochemical processes. Suppose that the sample is excited by *N_ex_* photons of energy *E_in_* and either decays radiationlessly to the ground state, or by photochemistry with a probability Φ*_P_* to a stable product state of energy *E_P_* above the ground state. The total heating *dQ* will then be given by

$$dQ=N_{ex}\left(E_{in}-\Phi_{P}E_{P}\right).$$ (11)

Equation (11) shows that energy levels of photochemical products can be obtained if the yields are known, or the yields can be determined if the energy levels are known. Also, the rates of formation of the photochemical products can be measured or deduced and finally, as Rosencwaig has pointed out [17], the excitation spectrum of the photochemical process can be obtained, and compared with the conventional absorption spectrum.

The work of deGroot et al. [24] illustrates the use of the acoustic spectrophone to study photochemical processes of acetaldehyde. These authors find that at pressures below a few torr the optoacoustic spectrum in the region 230–360 nm resembles the normal absorption spectrum of the compound. When the pressure is increased, however, a minimum appears in the spectrum at 290 nm which indicates that at least some of the absorbed light energy is no longer converted to heat on the time scale of chopping. The authors ascribe this phenomenon to the efficient formation of a high energy unstable intermediate, a dioxetene. Studies with a pulsed light source shows that the intermediate decays to stable low energy products with a time constant of 2 ms. At wavelengths shorter than 290 nm the heating is again rapid; this is ascribed to the rapid decomposition of vibrationally hot dioxetene to low energy products.

Another interesting study using the acoustic spectrophone has been reported by Kaya, Harshbarger and Robin [25]. These investigators observed the gas phase optoacoustic excitation spectrum of biacetyl under a wide variety of conditions. They were able to demonstrate that the lowest excited triplet could be populated only by excitation at wavelengths longer than 443 nm; irradiation at shorter wavelengths resulted in intersystem crossing to the second excited triplet which then decayed rapidly and directly to the ground state. In mixtures of benzene and biacetyl, the authors were able to show that energy transfer from benzene to the biacetyl triplet manifold takes place only when exciting into the lowest vibronic bands of the lowest excited singlet of the donor. In pyridine- biacetyl mixtures, the lowest n – π* state of pyridine was found to transfer to the triplet manifold of biacetyl, but the lowest π – π* state does not.

A final study by Callis, Parson and Gouterman [26] illustrates the potential application of calorimetric techniques to the study of photobiological systems. A very useful model system for the study of the bioenergetics of photosynthesis is the chromatophore of photosynthetic bacteria. The chromatophores are vesicular fragments of the bacterial membrane, which contain the photosensitive pigments and most of the enzymes necessary for light induced electron transport and coupled phosphorylation. In the chromatophore light induces a cyclic electron flow which is coupled in some unknown manner to the formation of high energy phosphate bonds, in which the free energy available from the photons are stored.

In the study of photochemical reactions in the liquid and solid phases using a volume transducer we must include the possibility of a volume difference Δ*V_r_* between reactants and products, as well as a contribution from heating. For a system which either returns radiationlessly to the ground state or converts to a product state *E_p_* with efficiency Φ*_p_* the total volume change Δ*V* will be

$$\Delta V=N_{ex}\left(E_{in}-\Phi_{p}E_{p}\right)\left(\alpha /\rho C_{p}\right)+N_{ex}\Phi_{p}\Delta V_{r}.$$ (12)

If *E_p_* and Δ*V_r_* are temperature independent, the measurement of Δ*V* at two different temperatures allows one to obtain *E_p_* and Δ*V_r_*.

**Figure 5:**
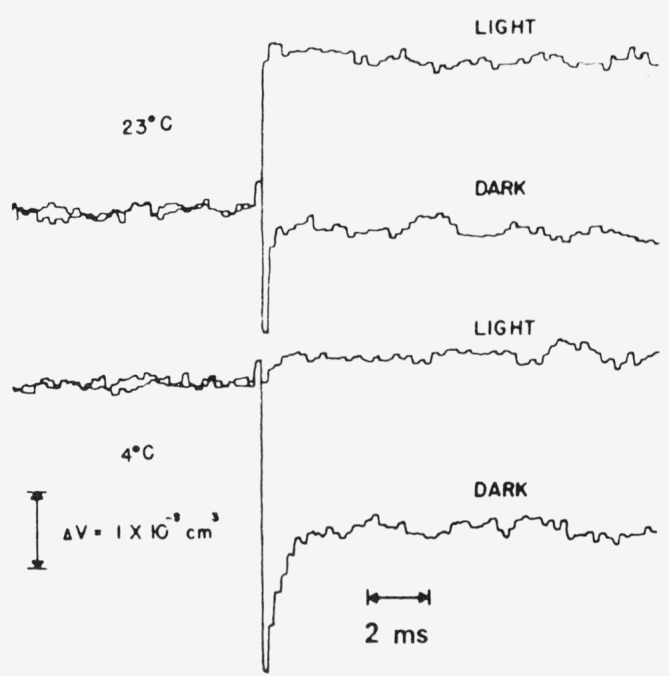
Volume changes of Chromatium chromatophores in response to flash excitation.

Figure 5 shows the volume response of *Chromatium* chromatophores to weak flashes at temperatures of 23°C and 4°C. The traces labeled “light” were obtained in the presence of a strong cw light which saturated all photochemical processes, and resulted in the conversion of all of the input energy to heat. The light response at 4°C is much smaller due to the reduced magnitude of *α*/*ρC_p_* at this temperature. In the dark at 23 °C, a weak flash causes an instantaneous volume decrease which is then followed by a fast recovery almost to the base line. In the dark at 4 °C we observe an instantaneous volume decrease which recovers about half way with a time constant of 250 *μs.* From a quantitative evaluation of these data, we have concluded that: (a) the initial high energy state of the photosynthetic apparatus does not possess a significant enthalpy change from the ground state, and thus the free energy change available from the photon is stored in an excited state characterized by negative entropy; (b) the high energy state also has a decreased volume from the ground state. Further studies have shown that the volume changes are altered by the presence of ion transporting antibiotics, and uncouplers of phosphorylation. The flash calorimeter thus appears to be a valuable tool for the study of energy conservation in photosynthetic systems.

## 4. Conclusions

Calorimetric techniques can be used to measure a number of parameters of excited states such as lifetimes, excitation spectra, energy yields of radiationless and photochemical processes, and enthalpies of photochemical reactions. Simple calorimeters capable of measuring these parameters have been developed for the study of molecules in the gas, solid and liquid phases. A wide variety of measurements now exist which show the usefulness of calorimetric techniques for studies of absolute fluorescence quantum efficiencies, quantum yields of triplet formation, photochemical processes in the gas phase, and for studies of energy storage in photosynthesis. As more investigators realize how simple and convenient these measurements are, we can expect an increase in the use of calorimetric techniques in photophysical and photochemical studies.

## Footnotes

1 Figures in brackets indicate the literature references at the end of this paper.

2 In order to adequately describe materials and experimental procedures, it is occasionally necessary to identify commercial products by manufacturer’s name or label. In no instance does such identification imply endorsement by the National Bureau of Standards, nor does it imply that the particular product or equipment is necessarily the best available for that purpose.
